# Impact of Pre‐Operative Nutritional Status on Postoperative Outcomes Following Pelvic Exenteration: A Systematic Review and Meta‐Analysis

**DOI:** 10.1111/ans.70896

**Published:** 2026-08-20

**Authors:** Lorcan Byrne, Cian Keogh, Craig Harris, Eamon Francis, Christina Buckley, Peter Lonergan, Louise McLoughlin, Feras Abu Saadeh, Michael Kelly, Ben Creavin

**Affiliations:** ^1^ Trinity St. James's Cancer Institute, St. James's Hospital Dublin Ireland; ^2^ Department of Surgery Royal Brisbane and Women's Hospital Brisbane Australia; ^3^ Department of Surgery University of Dublin – Trinity College Dublin Dublin Ireland

**Keywords:** body mass index, malnutrition, pelvic exenteration, postoperative complications, preoperative assessment, serum albumin

## Abstract

**Background:**

Pelvic exenteration is an extensive procedure for advanced or recurrent pelvic malignancies, associated with high morbidity and mortality. Malnutrition is common in this cohort and may affect postoperative outcomes, but the current evidence remains inconsistent.

**Materials and Methods:**

A systematic review was performed following PRISMA guidelines. Pooled analyses were used to assess the relationship between preoperative nutritional status (serum albumin, BMI, and Subjective Global Assessment (SGA)) and postoperative outcomes, with results shown as odds ratios (ORs) and 95% confidence intervals (CIs).

**Results:**

Nine retrospective cohorts comprising 1057 patients were included. The median age across studies was 56 years. Primary tumour types included colorectal, gynaecological, bladder, and soft tissue sarcoma. All studies reported complications, most of which were classified using the Clavien–Dindo system. Low BMI was linked to increased postoperative morbidity (OR 1.12, 95% CI 1.02–1.23, *p = 0.02). Hypoalbuminemia (< 35 g/L) (OR 1.38, 95% CI 0.62–3.08, *p* = 0.44) and SGA‐defined malnutrition (OR 1.24, 95% CI 0.24–6.48, *p* = 0.09) showed non‐significant but consistent trends towards higher complications in malnourished patients. Secondary outcomes were statistically significant, with malnourished patients experiencing longer hospital stays (SMD 0.44, 95% CI 0.26–0.62, *p < 0.00001) and more reoperations (OR 1.82, 95% CI 1.08–3.09, *p = 0.03).

**Conclusion:**

Pre‐operative malnutrition is linked to increased morbidity after pelvic exenteration. Low BMI was associated with increased postoperative morbidity following pelvic exenteration, while hypoalbuminaemia and SGA‐defined malnutrition demonstrated consistent but non‐significant associations. These findings support routine nutritional screening and preoperative optimisation in this high‐risk patient group.

## Graphical abstract

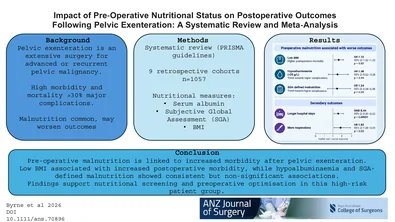

## 1 Introduction

Pelvic exenteration involves *en‐bloc*, multi‐visceral removal of pelvic organs typically performed for advanced or recurrent pelvic cancers. It is usually reserved for patients with limited alternative treatment options [1, 2, 3]. While pelvic exenteration can extend survival and, in some cases, offer a chance for cure, it is linked to significant morbidity, mortality, and impacts on quality of life [4, 5, 6, 7]. Reported major complication rates can be over 30%, including wound infection, anastomotic leaks, and sepsis [7]. Many patients require extended hospital stays and intensive postoperative care. This morbidity has sparked growing interest in modifiable patient‐related factors that might improve outcomes.

Malnutrition is highly common among patients undergoing major oncological surgery and is a recognised predictor of poor outcomes, including higher rates of infection, impaired wound healing, longer hospital stays, and increased mortality [8]. Patients considered for pelvic exenteration are especially vulnerable to nutritional issues due to the combination of advanced disease, previous chemoradiotherapy, and chronic tumour‐related metabolic stress [5]. These factors often lead to weight loss, sarcopenia, and functional decline before surgery.

Preoperative nutritional assessment is increasingly recognised as a vital part of surgical prehabilitation [9, 10]. Studies have shown that interventions, such as prehabilitation, aimed at malnourished patients can reduce complications, shorten hospital stays, and enhance tolerance to adjuvant therapies [9, 10]. However, there is no single validated assessment tool for evaluating preoperative nutritional status in surgical patients [11]. Commonly used tools include body mass index (BMI), serum albumin, Subjective Global Assessment (SGA)—an evaluation of a patient's history and physical examination using structured clinical parameters to diagnose malnutrition—and CT‐based body composition analysis (sarcopenia), but each has limitations. Consequently, the effect of preoperative malnutrition on pelvic exenteration outcomes remains uncertain.

Considering the high complexity of pelvic exenteration and patients' vulnerability to malnutrition [12], understanding the impact of preoperative nutrition is clinically crucial. This systematic review and meta‐analysis aimed to assess the relationship between preoperative nutritional status and postoperative complications after pelvic exenteration, to determine whether preoperative malnutrition predicts poorer outcomes, and to guide preoperative optimisation strategies.

## 2 Materials and Methods

### 2.1 Study Design

This systematic review was conducted following the PRISMA guidelines [13]. The main research question was whether poor preoperative nutritional status is linked to higher postoperative complications after pelvic exenteration surgery.

### 2.2 Search Strategy

A comprehensive electronic search was undertaken across Scopus, MEDLINE, Embase and PubMed. The final search was conducted on 1st May 2025. A comprehensive search was carried out by combining the following search terms with AND/OR Boolean operators: (pelvic exenteration) AND (nutrition OR nutritional OR malnutrition OR nutritional status) AND (preoperative OR perioperative) AND (complications OR morbidity OR mortality) AND (outcome OR prognosis OR survival). The search was restricted to studies published in English from January 2000 onwards. All duplicate records were identified and manually removed prior to screening. Additionally, included articles' references were screened for any relevant publications for inclusion.

### 2.3 Eligibility Criteria

Studies were included if they were retrospective clinical cohort designs reporting on both preoperative nutritional status and postoperative outcomes in patients undergoing pelvic exenteration. To be eligible, studies were required to employ an explicit measure of preoperative nutritional status. Studies were excluded if they were not published in English, if they failed to provide preoperative nutritional assessment, or if they did not report on patient outcomes.

### 2.4 Study Selection

Study selection was performed using Rayaan systematic review tool (https://www.rayyan.ai). Titles and abstracts were first screened for potential eligibility, and the full texts of studies considered relevant were subsequently retrieved and assessed against the established inclusion criteria. Compliance with the criteria was confirmed for each study, and selection was confirmed by two reviewers.

### 2.5 Data Extraction

Data extraction followed using a standardised process and included author, year of publication, country of origin, study design, and sample size. Patient demographics, details of nutritional assessment, and reported outcomes were also recorded. The primary outcome of interest was the occurrence of postoperative complications. Secondary outcomes included length of hospital stay, return to theatre, survival, and post‐operative quality of life (QOL).

### 2.6 Quality Assessment

The methodological quality of included studies was assessed using the Newcastle–Ottawa Scale (NOS) for cohort studies [14]. This tool scores quality across three domains: selection of study groups (maximum 4 points), comparability of cohorts (maximum 2 points), and assessment of outcomes (maximum 3 points), with a maximum score of 9. Studies scoring 8–9 were considered high quality, those scoring 6–7 moderate quality, and those scoring ≤ 5 were considered low quality; see Table 1.

**TABLE 1:** NOS quality assessment.

Study	Selection	Comparability	Outcome	Total
Beaton et al. 2014	3	1	2	6
Lyell et al. 2019	4	2	3	9
Bacalbasa et al. 2019	3	1	2	6
Tortorella et al. 2024	4	2	3	9
Hogan et al. 2021	4	2	3	9
Seebacher et al. 2022	3	1	2	6
van Rees et al. 2021	4	2	3	9
Bedrikovetski et al. 2023	4	1	2	7
Watt et al. 2024	4	2	3	9

### 2.7 Statistical Analysis

Statistical analysis was performed using Review Manager version 5.3 (The Nordic Cochrane Centre, The Cochrane Collaboration, Copenhagen, Denmark). Dichotomous data from papers were converted to a Log Odds Ratio with standard error and analysed using generic inverse variance. For continuous data, standard mean difference (SMD) and 95% CI were estimated using inverse‐variance weighting. Outcome measures were recorded as mean (s.d) based on available data. Effect estimates were interpreted according to the direction of comparison defined in each individual analysis. Heterogeneity was assessed using the I^2^ statistic, with values > 50% indicating substantial heterogeneity. Pooled estimates of differences were calculated using random‐effects models, accounting for potential interstudy heterogeneity. Sensitivity analyses were carried out where appropriate. *p* value < 0.05 was considered statistically significant.

## 3 Results

**FIGURE 1:**
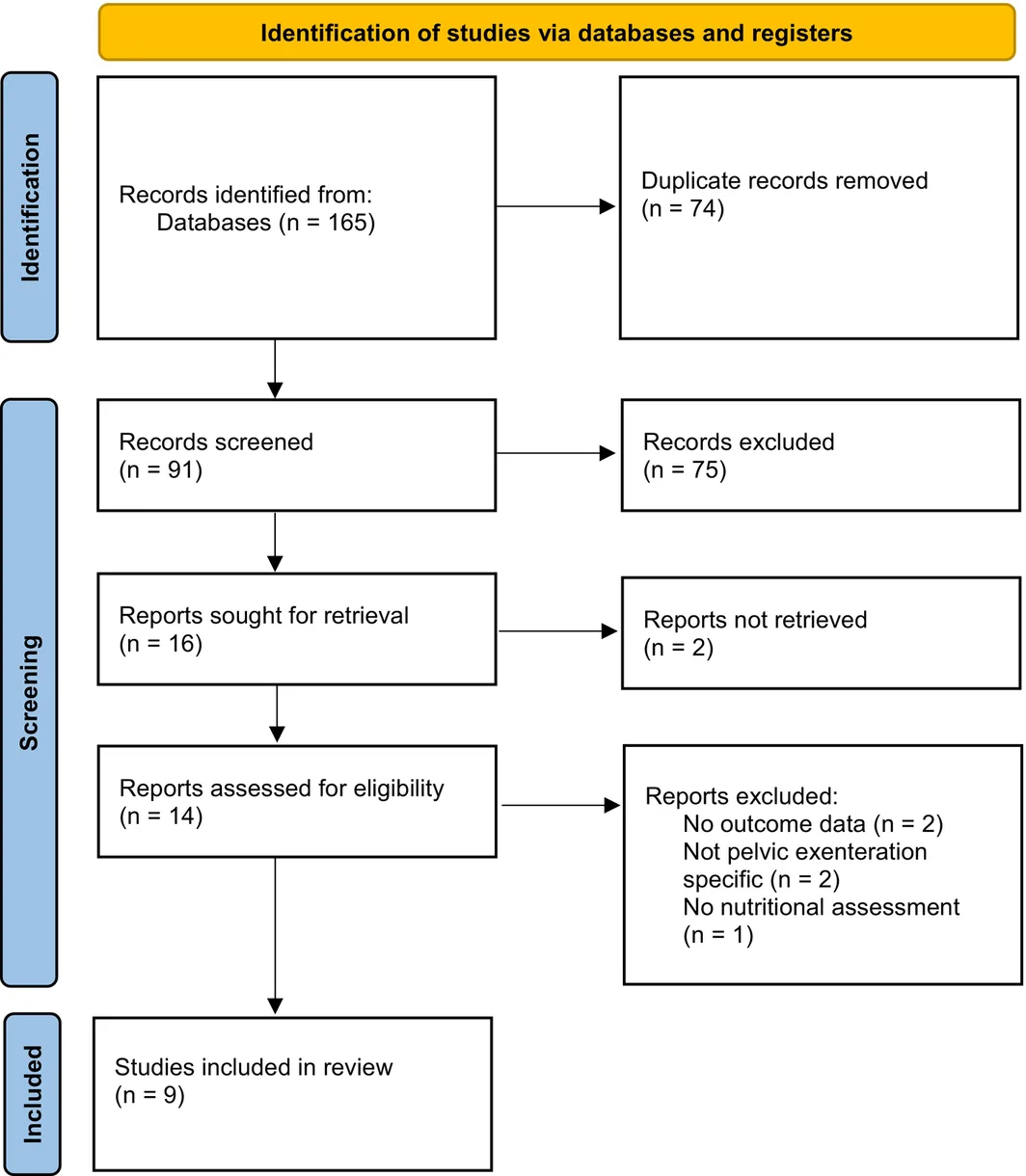
PRISMA flow chart demonstrating study selection and screening process.

A total of 165 records were initially identified through database searches. Before screening, 74 duplicate records were removed. This left 91 articles for abstract screening; 75 articles were excluded after this step. A total of 16 articles were sought for retrieval, but two could not be retrieved. After full‐text screening, five articles were excluded. Ultimately, nine studies were included in this systematic review [15, 16, 17, 18, 19, 20, 21, 22, 23]. The selection process is detailed in the PRISMA flow diagram (Figure 1).

### 3.1 Study Characteristics

A total of nine retrospective cohort studies published between 2014 and 2024 were included [15, 16, 17, 18, 19, 20, 21, 22, 23], comprising 1057 patients undergoing pelvic exenteration for advanced pelvic malignancy. Sample sizes ranged from 31–227 patients. Most studies were single institutions, although one included a multicentre cohort [16].

Nutritional status was measured using a range of tools—anthropometric measures such as body mass index (BMI) [15, 18, 20, 21, 23], biochemical markers including serum albumin [16, 17, 18, 20, 21, 22], validated screening/assessment tools such as the Subjective Global Assessment (SGA) [19, 23, 24] and Malnutrition Universal Screening Tool (MUST) [21] and CT‐based body composition analysis (sarcopenia) [18, 20, 21, 22].

All studies reported postoperative complications, with definitions mostly based on the Clavien–Dindo classification [25]. Several also examined secondary outcomes such as length of stay (LOS), readmission/reoperation rates, quality of life (QOL), and overall survival.

### 3.2 Patient Demographics

Across the nine included studies, a total of 1057 patients underwent pelvic exenteration. Median age at surgery across the studies was 56 years. Several series reported exclusively female cohorts, reflecting inclusion of gynaecological malignancies [17, 18, 20]. When pooled, the overall sex distribution was 72.7% female and 27.3% male.

Primary indications for exenteration varied across studies. The majority of patients had locally advanced or recurrent rectal cancer or gynaecological cancers (cervical, endometrial, ovarian, vaginal). Smaller numbers of patients with bladder tumours and pelvic soft tissue sarcomas were also included in mixed cohorts. Study demographics are summarised in Table 2.

**TABLE 2:** Study demographics.

Author	Sample size	Study design	Nutritional assessment	Outcomes
Beaton et al. 2014	31	Retrospective cohort	BMI	Complications, LOS
Lyell et al. 2019	199	Retrospective cohort	Serum albumin	Complications, LOS, readmission, reoperation, survival
Bacalbasa et al. 2019	86	Retrospective cohort	Serum albumin	Complications, LOS, survival
Tortorella et al. 2024	129	Retrospective cohort	BMI, serum albumin, sarcopenia	Complications
Hogan et al. 2021	123	Retrospective cohort	SGA	Complications, LOS, re‐operation
Seebacher et al. 2022	65	Retrospective cohort	BMI, serum albumin, sarcopenia	Complications, survival
Van Rees et al. 2021	227	Retrospective cohort	BMI, serum albumin, sarcopenia	Complications
Bedrikovetski et al. 2023	128	Retrospective cohort	Serum albumin, sarcopenia	Complications, LOS, re‐admission, survival
Watt et al. 2024	69	Retrospective cohort	BMI, SGA	Complications

### 3.3 Quality Assessment

The methodological quality of included studies was assessed using the Newcastle–Ottawa Scale (NOS) for cohort studies [14]. This tool scores quality across three domains: selection of study groups (maximum 4 points), comparability of cohorts (maximum 2 points), and assessment of outcomes (maximum 3 points), with a maximum score of 9. Studies scoring 8–9 were considered high quality, those scoring 6–7 moderate quality, and those scoring ≤ 5 were considered low quality. All included studies achieved greater than 6 points indicating moderate to high quality, see Table 1.

### 3.4 Serum Albumin

**FIGURE 2:**
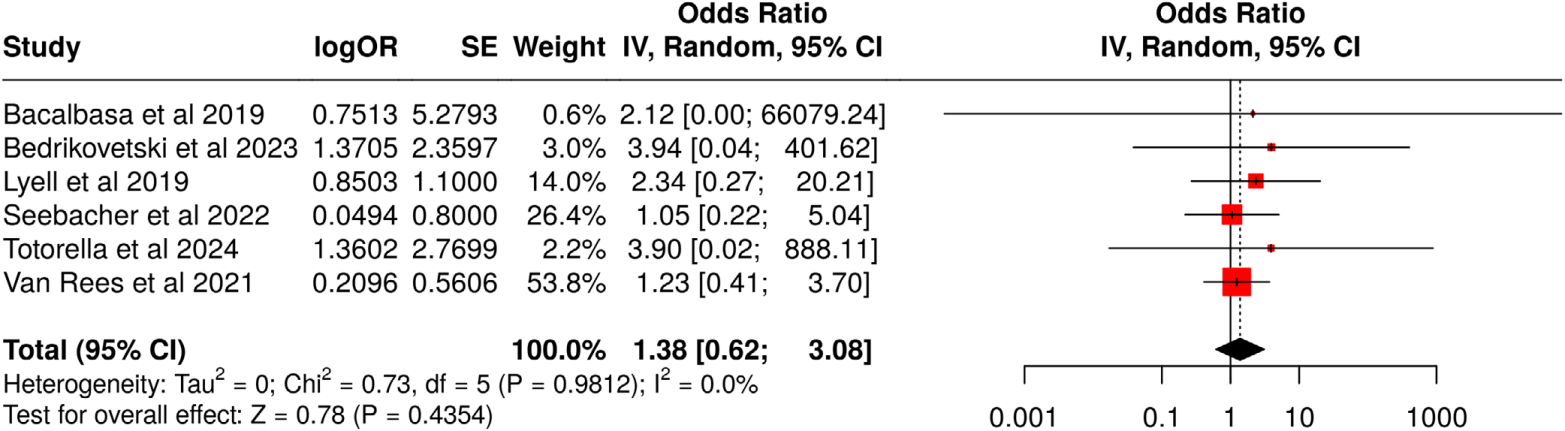
Forest plot demonstrating association between serum albumin and postoperative morbidity following pelvic exenteration.

Hypoalbuminaemia (< 35 g/L) was consistently associated with increased morbidity across the included studies. The pooled analysis shown in Figure 2 also suggested increased morbidity in hypoalbuminaemic patients; however, the association did not reach statistical significance (OR 1.38, 95% CI 0.62–3.08, *p* = 0.44, I^2^ = 0%).

### 3.5 Body Mass Index (BMI)

**FIGURE 3:**
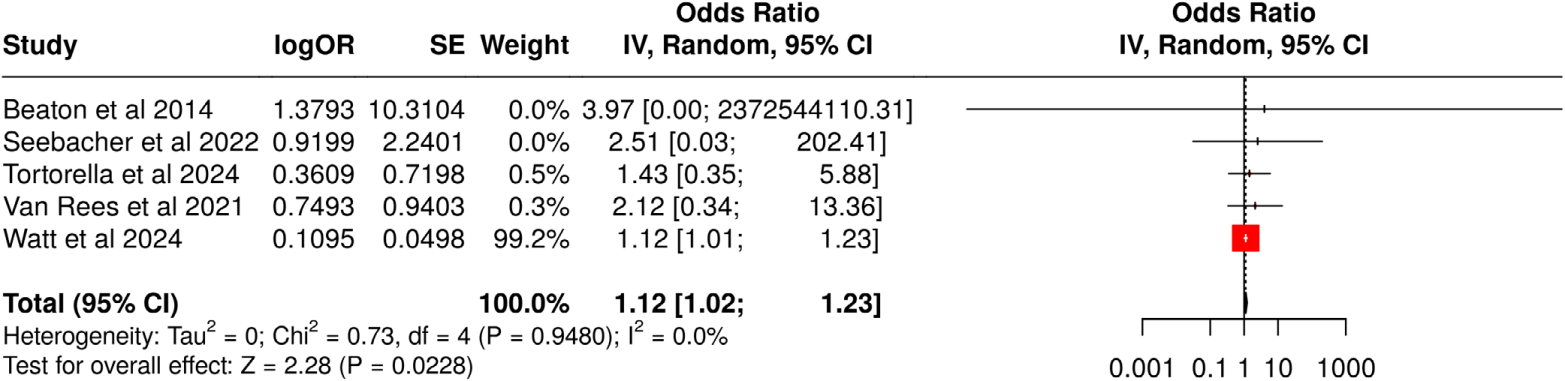
Forest plot demonstrating association between BMI and postoperative morbidity following pelvic exenteration.

Lower‐BMI patients were at greater risk of complications than those with normal or higher BMI. The pooled analysis in Figure 3 demonstrated a statistically significant association, with low BMI associated with increased postoperative morbidity. (OR 1.12, 95% CI 1.02–1.23, *p = 0.02, I^2^ = 0%).

### 3.6 Subjective Global Assessment (SGA)

**FIGURE 4:**
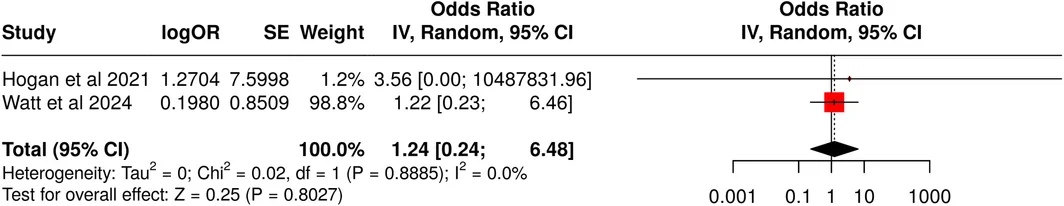
Forest plot demonstrating association between SGA and postoperative morbidity following pelvic exenteration.

Malnutrition, defined by Subjective Global Assessment (SGA B/C), was associated with higher complication rates in individual studies. The pooled analysis in Figure 4 showed a trend towards increased morbidity, but this did not reach statistical significance (OR 1.24, 95% CI 0.24–6.48, *p* = 0.09, I^2^ = 0%).

### 3.7 Return to Theatre

**FIGURE 5:**
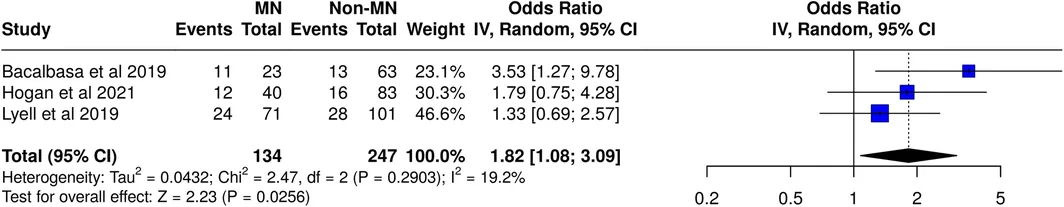
Forest plot demonstrating association between malnutrition and return to theatre following pelvic exenteration.

Pooled analysis in Figure 5 of return to theatre across all nutritional assessments showed a statistically significant difference between patient groups, with malnourished patients having a substantially higher rate of return to theatre than non‐malnourished patients. (OR 1.82, 95% CI 1.08–3.09, *p = 0.03, I^2^ = 19%).

### 3.8 Length of Stay

**FIGURE 6:**
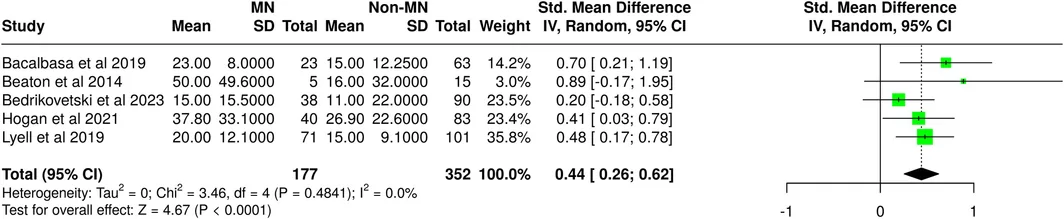
Forest plot demonstrating association between malnutrition and length of stay following pelvic exenteration.

Pooled analysis in Figure 6 of length of stay across all nutritional assessments also showed a statistically significant difference. Length of stay was significantly longer in malnourished patients compared to non‐malnourished patients. (SMD 0.44, 95% CI 0.26–0.62, *p < 0.00001, I^2^ = 0%).

## 4 Discussion

This systematic review and meta‐analysis demonstrated the impact of preoperative nutritional status on postoperative outcomes after pelvic exenteration. Across nine retrospective cohort studies involving more than 1000 patients, malnutrition, whether defined by anthropometry, biochemical markers, or validated screening tools, showed consistent trends towards higher morbidity. The meta‐analysis revealed a statistically significant association between low BMI and postoperative complications, while hypoalbuminemia and SGA‐defined malnutrition showed consistent but non‐significant trends. These findings suggest that impaired preoperative nutritional status may be associated with poorer postoperative outcomes following pelvic exenteration.

Our pooled analysis found that low BMI was the only nutritional measure significantly linked to postoperative complications. This aligns with previous data where low BMI was associated with impaired wound healing, increased infectious complications, and higher perioperative risk [26, 27]. Notably, the relationship between BMI and outcomes appears to be non‐linear. While underweight patients seem most vulnerable, obesity has also been identified as a risk factor in some of the included cohorts [21, 23]. Collectively, these observations suggest a *U*‐shaped association between BMI and postoperative morbidity, consistent with findings in broader oncological surgery populations [28].

Although hypoalbuminaemia did not show a significant overall effect, the trend was consistent across all studies, with several reporting significant findings individually [16, 17, 18]. These findings support the use of serum albumin as a marker of physiological reserve, while recognising that it is influenced by factors beyond nutritional status alone. The lack of a statistically significant pooled effect may be due to slight differences in definitions across studies (e.g., < 34 g/L vs. < 35 g/L), as well as the fact that hypoalbuminaemia may not reflect only nutritional deficiency but also systemic inflammation and advanced disease burden [29, 30]. Similarly, SGA was highly predictive in individual studies [19, 23], but the small number of cohorts prevented achieving statistical significance in the meta‐analysis. Importantly, both albumin and SGA are inexpensive, reproducible, and feasible for routine preoperative assessment. The observed increase in reoperation rates and longer hospital stays among malnourished patients also highlight the long‐term impact of preoperative nutritional status beyond the immediate post‐operative period.

It is important to recognise that BMI, serum albumin and SGA assess different aspects of nutritional and physiological status. BMI measures body mass relative to height but does not distinguish between lean muscle mass and adiposity. Serum albumin is influenced by nutritional status but also by systemic inflammation and disease burden, making it a marker of physiological reserve rather than nutrition alone [29, 30]. In contrast, SGA incorporates clinical history and physical examination findings to provide a more comprehensive assessment of nutritional status. Consequently, these measures are not interchangeable, highlighting the importance of a comprehensive nutritional assessment rather than relying on a single marker to identify patients who may benefit from preoperative optimisation.

The high overall morbidity associated with pelvic exenteration highlights the importance of preoperative optimisation. Our findings support routine preoperative nutritional assessment in this group. These results align with broader oncological evidence, where international guidelines like the European Society for Clinical Nutrition and Metabolism (ESPEN) strongly advocate for routine nutritional screening and intervention in cancer patients undergoing major surgery [12]. Therefore, nutritional status should be considered in risk stratification tools, prehabilitation protocols, and preoperative patient counselling.

Contemporary prehabilitation programmes recognise that nutritional impairment rarely occurs in isolation. Malnutrition frequently coexists with sarcopenia, frailty and reduced functional capacity, all of which contribute to reduced physiological reserve before major surgery. Consequently, nutritional assessment should be viewed not simply as perioperative risk prediction, but as an opportunity to identify patients who may benefit from multimodal prehabilitation incorporating nutritional optimisation, exercise training and psychological support. Importantly, preoperative nutritional interventions, including dietetic optimisation and dietary supplementation, are feasible and have shown benefits in other major oncological surgeries [10, 31]. Our findings support implementing standardised preoperative nutritional assessment as both a method of identifying high‐risk patients and an opportunity to deliver targeted preoperative interventions.

This study has some limitations that should be considered when interpreting the findings. First, all included studies were retrospective cohort studies and are therefore subject to selection bias, confounding, and data‐collection limitations. The predominance of female patients across included cohorts may also limit the transferability of findings to male populations. Second, the relatively small number of included studies and moderate sample sizes reduce the statistical power of the meta‐analysis. This may explain why several consistent trends did not reach statistical significance despite agreement across studies.

Additionally, there was no standardised definition or assessment of preoperative nutritional status. Measures such as BMI, serum albumin, and SGA reflect different aspects of nutritional and physiological health and are not directly interchangeable. Variations in cut‐off values and assessment methods may have decreased comparability between studies. This was also true for outcome reporting, which was not standardised. Although most studies used the Clavien–Dindo classification, differences in reporting secondary outcomes introduce potential reporting bias.

Despite these limitations, this study provides a comprehensive synthesis of the available evidence and demonstrates consistent trends supporting an association between impaired preoperative nutritional status and poorer postoperative outcomes following pelvic exenteration.

## 5 Conclusion

This review provides a comprehensive synthesis of the available evidence and highlights the importance of preoperative nutritional status in influencing postoperative outcomes following pelvic exenteration. Our findings support routine preoperative nutritional assessment as part of preoperative optimisation in patients undergoing pelvic exenteration and reinforce the role of prehabilitation in this high‐risk cohort. Future prospective studies should evaluate comprehensive prehabilitation strategies incorporating nutritional assessment, body composition analysis, and functional performance measures to determine which combination of factors best predicts postoperative outcomes and identifies patients most likely to benefit from targeted intervention.

## Author Contributions

**Lorcan Byrne:** conceptualization, investigation, writing – original draft, methodology, writing – review and editing, software, formal analysis, data curation. **Christina Buckley:** writing – review and editing. **Louise McLoughlin:** writing – review and editing. **Michael Kelly:** writing – review and editing, supervision, conceptualization, project administration. **Peter Lonergan:** writing – review and editing. **Craig Harris:** writing – review and editing. **Eamon Francis:** writing – review and editing. **Feras Abu Saadeh:** writing – review and editing. **Ben Creavin:** writing – review and editing, conceptualization, investigation, methodology, formal analysis, supervision, data curation, project administration. **Cian Keogh:** writing – review and editing.

## Funding

The authors have nothing to report.

## Conflicts of Interest

The authors declare no conflicts of interest.

## Data Availability

Data discussed in this article will be made available by the authors upon reasonable request.
